# A 14‐Year‐Old Male With Susac Syndrome: A Case Report

**DOI:** 10.1155/crnm/9280625

**Published:** 2026-06-18

**Authors:** M-Jaber Kojak, Mouhammed Sleiay, Bilal Sleiay, Abdulrahman Dokhan, Ghassan Hamzeh, Hasan Alsmoudi, Bara’a Merie Alghadi

**Affiliations:** ^1^ Department of Internal Medicine, Damascus University, Damascus, Syria, damascusuniversity.edu.sy; ^2^ Faculty of Medicine, Hama University, Hama, Syria; ^3^ Department of Neurology, National University Hospital, Damascus, Syria, nuh.com.sg; ^4^ Orthopedic Surgery Department, Ibn Al-Nafees Hospital, Damascus, Syria; ^5^ Faculty of Medicine, Yarmouk University, Irbid, Jordan, yu.edu.jo

**Keywords:** case report, cerebral vasculitis, fluorescein angiography, Susac syndrome

## Abstract

**Introduction and Importance:**

Susac syndrome (SuS) is a rare autoimmune microangiopathy involving the brain, retina, and inner ear. It is uncommon in children, and early diagnosis is essential to prevent irreversible deficits.

**Case Presentation:**

A 14‐year‐old boy presented with decreased consciousness following 18 days of excessive sleepiness, vomiting, blurred vision, and unsteady gait. There was no family history of autoimmune diseases. MRI showed “snowball‐like” corpus callosum lesions suggestive of SuS. Cerebrospinal fluid (CSF) revealed lymphocytic pleocytosis and elevated protein. He improved partially with intravenous methylprednisolone but later developed status epilepticus, requiring plasma exchange. During steroid taper, he experienced relapses with visual and gait disturbances. Fluorescein angiography (FA) confirmed branch retinal artery occlusions (BRAOs). Immunosuppressive therapy with azathioprine and rituximab was initiated, achieving disease stability.

**Clinical Discussion:**

MRI findings of corpus callosum “snowball” lesions are a key clue for early diagnosis. Multimodal management—including corticosteroids, plasma exchange, and long‐term immunosuppression—is essential to control relapses and prevent permanent sequelae. The patient’s fluctuating course underscores the need for ongoing monitoring.

**Conclusion:**

The identification of snowball lesions in the corpus callosum, in conjunction with a “string of pearls” appearance in the internal capsule, mandates prompt immunotherapeutic intervention. Rituximab is currently regarded as the preferred agent, given that azathioprine has demonstrated limited efficacy in moderating disease activity.

## 1. Introduction

Susac syndrome (SuS) is a rare autoimmune microangiopathy affecting the brain, retina, and inner ear [1]. It is characterized by the clinical triad of encephalopathy, branch retinal artery occlusion (BRAO), and low‐frequency sensorineural hearing loss, along with typical “snowball” lesions in the corpus callosum on magnetic resonance imaging (MRI) [2]. Disease severity ranges from mild reversible ischemia to irreversible microinfarction. SuS predominantly affects young women aged 20–40 years, and pediatric cases are extremely rare. Diagnosis is often delayed because symptoms may appear sequentially and mimic other conditions such as multiple sclerosis, Ménière’s disease, or cerebral vasculitis [3]. MRI, fluorescein angiography (FA), and audiometry are essential for diagnosis [4]. Treatment generally includes corticosteroids, intravenous immunoglobulin, and immunosuppressants such as azathioprine or cyclophosphamide, while rituximab is reserved for refractory cases [5]. We report a rare case of SuS in a 14‐year‐old boy, one of the youngest reported male patients.

## 2. Case Presentation

A 14‐year‐old previously healthy boy presented to our emergency department with decreased level of consciousness. Symptoms had begun 18 days earlier with excessive sleepiness, followed by vomiting that improved with ondansetron. Shortly thereafter, he developed blurred vision in the right eye and an unsteady gait. Progressively, he became mute and unresponsive to verbal commands. His mother reported a brief febrile episode at symptom onset. There was no family history of autoimmune diseases.

On examination, his Glasgow Coma Scale was 9 (E2V2M5). Pupils were equal and reactive, but the oculocephalic reflex was absent on leftward rotation. Other brainstem reflexes were intact. Motor examination showed normal tone, symmetrical withdrawal to pain, hyperreflexia, and bilateral extensor plantar responses. No meningeal signs were present.

Initial laboratory investigations were unremarkable. Cerebrospinal fluid (CSF) analysis revealed pleocytosis with a white blood cell count of 20/μL (predominantly lymphocytes) and elevated protein of 140 mg/dL. Brain MRI revealed multiple “snowball‐like” lesions in the corpus callosum (Figure 1) and a “string of pearls” appearance in the internal capsule (Figure 2), raising suspicion of SuS.

**FIGURE 1:**
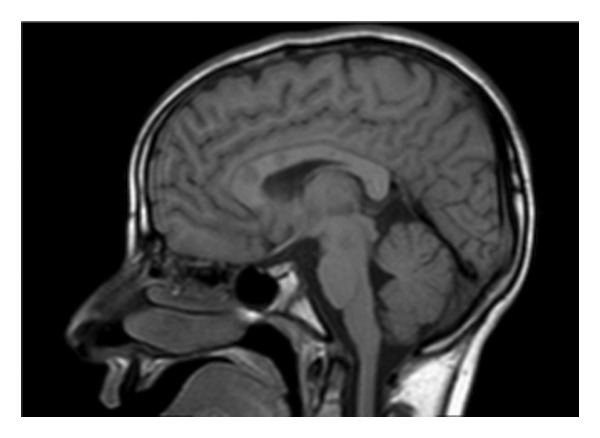
Brain MRI showing multiple “snowball‐like” lesions in the corpus callosum. These lesions appear hypointense on T1‐weighted imaging, characteristic of Susac syndrome.

**FIGURE 2:**
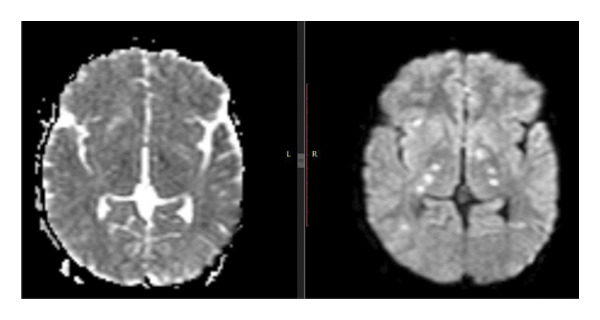
Brain MRI showing a “string of pearls” appearance in the internal capsule. The right image is diffusion‐weighted imaging (DWI) with small locunar hyperintensity, and the left one is apparent diffusion coefficient (ADC) with corresponding hypointensity in bilateral internal capsule, suggestive of locunar microinfarctions.

On the first day of hospitalization, the patient received intravenous methylprednisolone at a dose of 1 g/day for 5 days. He showed partial clinical improvement, becoming more responsive to simple commands, though significant neurological deficits persisted. On Day 6, one day after completing the steroid course, he developed generalized tonic‐clonic status epilepticus lasting approximately 45 min, requiring intubation and transfer to the intensive care unit. Status epilepticus was controlled within 20 min after administration of intravenous levetiracetam (loading dose of 60 mg/kg followed by 40 mg/kg/day) and a midazolam infusion (0.2 mg/kg bolus then 0.1 mg/kg/hour). Immediately thereafter, from Day 7 to Day 11, therapeutic plasma exchange (five cycles, one exchange daily) was initiated. The patient showed progressive recovery starting after the third plasma exchange, with gradual return of eye opening to command, extubation by Day 10, and ability to speak single words by discharge. Cervical spine MRI, FA, and audiogram were initially unremarkable. He was discharged on oral prednisolone (1 mg/kg/day) with a gradual taper over 6 weeks. Continuation of antiseizure medication for at least 1 month would have been ideal for this acute symptomatic status epilepticus, but the patient was not maintained on any antiepileptic drug after discharge, as the family could not afford the medications, and they were not obtainable locally. Mild residual gait instability persisted but improved with rehabilitation.

During steroid taper, the patient experienced recurrent relapses, including unsteady gait with cerebellar signs, and later tinnitus and blurred vision. Repeated brain MRI confirmed persistent callosal lesions, while FA revealed BRAO in the right eye (Figure 3). Audiometry remained normal. Given the relapsing course, azathioprine was introduced. Subsequently, rituximab was added in combination with corticosteroids due to further relapses. Follow‐up evaluations (brain MRI, FA, and audiogram) showed stable disease.

**FIGURE 3:**
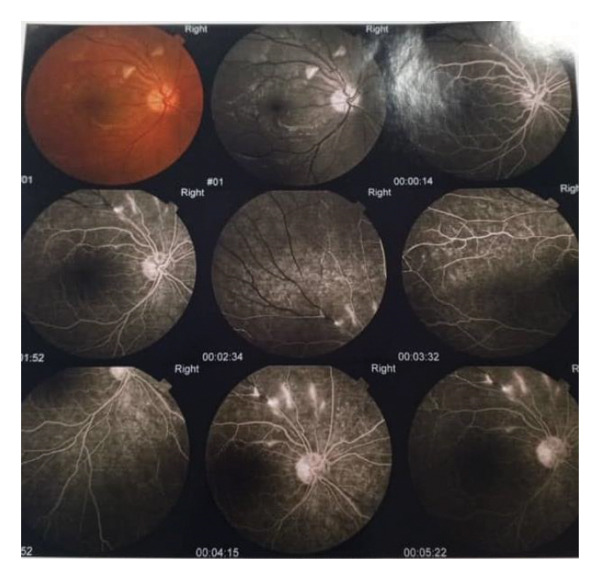
Fluorescein angiography (FA) demonstrating branch retinal artery occlusions (BRAO) in the right eye, confirming retinal involvement consistent with Susac syndrome.

## 3. Discussion

SuS is an uncommon immune‐mediated microangiopathy affecting the small arterioles of the brain, retina, and inner ear. The complete clinical triad comprises encephalopathy, BRAO, and sensorineural hearing loss [6]. The condition was first documented by Susac et al. in 1979 and later named by William F. Hoyt in 1986 [7]. SuS predominantly affects young women aged 20–40 years, with a female‐to‐male ratio of 3.5:1, and shows a preference for individuals of Caucasian descent [8, 9]. Pediatric cases are rare [10], with an annual incidence of approximately 0.024 per 100,000 (95% CI 0.010–0.047) [11]. Our case is unique as it describes a male patient of this age group with SuS.

The exact pathophysiology remains incompletely understood; proposed mechanisms include immune dysregulation, vasospastic phenomena, and coagulopathy. A notable hypothesis suggests that SuS may represent an autoimmune endotheliopathy or coagulopathy similar to catastrophic antiphospholipid syndrome. Past infections have been proposed as potential triggers, and antiendothelial cell antibodies have been detected in some patients, though no definitive evidence has confirmed a causal role [12].

Clinically, SuS is defined by encephalopathy, visual loss, and hearing impairment; however, this triad is not consistently present in all patients [7]. The full triad is identified in only 13%–30% of individuals at disease onset [1] (i.e., it is absent in 70%–87% of patients at presentation). Central nervous system manifestations are the most frequent initial feature [4], and the complete triad may take several months to 2 years to develop, leading to an average diagnostic delay of approximately 21 weeks [13]. In a large case series, the triad was eventually met in 85% of patients, while 15% remained incomplete [13]. In our case, the patient presented with altered consciousness, nausea, blurred vision in the right eye, unsteady gait, and eventually mutism; hearing loss and BRAO were confirmed later during follow‐up.

The differential diagnosis is broad and includes demyelinating diseases (e.g., multiple sclerosis or acute disseminated encephalomyelitis), autoimmune disorders (e.g., systemic lupus erythematosus or neuro‐Behçet disease), and vascular occlusions (e.g., embolic or atherosclerotic) [14]. Because of the variable presentation, several diagnostic criteria have been proposed, but SuS remains a clinical diagnosis, supported by ancillary tests when the triad is incomplete [8].

CSF analysis typically shows mild lymphocytic pleocytosis and elevated protein, as seen in our patient (WBC 20/μL, protein 140 mg/dL). Although nonspecific, CSF analysis helps narrow the differential diagnosis and exclude infectious etiologies [10].

Brain MRI is a cornerstone of diagnosis. It reveals white matter lesions in periventricular areas, the centrum semiovale, subcortical regions, and particularly the corpus callosum. Callosal lesions are nearly pathognomonic: they appear as small (3–7 mm), well‐defined T2/FLAIR hyperintensities (“snowball” lesions) that may coalesce into larger (> 7 mm) “wheel spoke” or “stalactite” formations. These lesions are hypointense on T1‐weighted imaging [15]. Importantly, SuS lesions typically involve the central fibers of the corpus callosum, whereas in multiple sclerosis and ADEM, lesions are located on the undersurface at the septal interface [16]. In our patient, brain MRI showed multiple “snowball” lesions in the corpus callosum and a “string of pearls” appearance in the internal capsule.

FA plays an essential role by demonstrating hyperfluorescence of affected retinal arteriolar walls, as well as BRAOs. Gass plaques (yellowish lesions from blood and lipid leakage) may also be observed but are neither specific nor pathognomonic [10]. MRI and FA remain the foundation of diagnostic evaluation [17]. In our case, FA confirmed BRAO in the right eye.

There are no standardized treatment protocols for SuS; therapy must be individualized. Intensive immunosuppression is recommended, especially when encephalopathy is present, because immediate, rigorous, and sustained intervention is vital. High‐dose corticosteroids are the empirical first‐line treatment, given the inflammatory nature of the disease. Other agents include azathioprine, mycophenolate mofetil, cyclophosphamide, and rituximab. Intravenous immunoglobulin is also used, often in combination with corticosteroids [10]. The natural history of SuS is variable; the disease may remain active for months, and remission can be prolonged. Most patients have some degrees of residual impairment at follow‐up [18]. In our case, initial treatment with methylprednisolone provided only partial improvement. After recurrent episodes of neurological symptoms with tinnitus and visual disturbances, we added azathioprine, and later rituximab, in combination with corticosteroids. The patient was subsequently monitored with MRI, FA, and audiometry, and his condition remained stable.

## 4. Conclusion

SuS is a rare autoimmune disease more common in females but can occasionally present in young males. Early diagnosis is challenging due to variable and sequential symptom onset. The presence of snowball lesions in the corpus callosum together with a string‐of‐pearls appearance in the internal capsule should prompt immediate immunotherapy. Rituximab is currently considered the treatment of choice in such cases, as azathioprine appears less effective in controlling disease activity.

## Funding

No funding was received for this research.

## Disclosure

The work has been reported in line with the SCARE criteria [19].

## Ethics Statement

The authors have nothing to report.

## Consent

Written informed consent was obtained from the patient/legal guardian for publication of this case report and any accompanying images and videos. A copy of the written consent is available for review by the Editor‐in‐Chief of this journal on request.

## Conflicts of Interest

The authors declare no conflicts of interest.

## Data Availability

Data sharing is not applicable to this article as no datasets were generated or analyzed during the current study.
